# NF-YAl drives EMT in Claudin^low^ tumours

**DOI:** 10.1038/s41419-023-05591-9

**Published:** 2023-01-28

**Authors:** Michela Londero, Alberto Gallo, Camilla Cattaneo, Anna Ghilardi, Mirko Ronzio, Luca Del Giacco, Roberto Mantovani, Diletta Dolfini

**Affiliations:** Dipartimento di Bioscienze, Università degli Studi di Milano, Via Celoria 26, 20133 Milano, Italy

**Keywords:** Tumour biomarkers, Breast cancer, Gastric cancer

## Abstract

NF-Y is a trimeric transcription factor whose binding site -the CCAAT box- is enriched in cancer-promoting genes. The regulatory subunit, the sequence-specificity conferring NF-YA, comes in two major isoforms, NF-YA long (NF-YAl) and short (NF-YAs). Extensive expression analysis in epithelial cancers determined two features: widespread overexpression and changes in NF-YAl/NF-YAs ratios (NF-YAr) in tumours with EMT features. We performed wet and in silico experiments to explore the role of the isoforms in breast -BRCA- and gastric -STAD- cancers. We generated clones of two Claudin^low^ BRCA lines SUM159PT and BT549 ablated of exon-3, thus shifting expression from NF-YAl to NF-YAs. Edited clones show normal growth but reduced migratory capacities in vitro and ability to metastatize in vivo. Using TCGA, including upon deconvolution of scRNA-seq data, we formalize the clinical importance of high NF-YAr, associated to EMT genes and cell populations. We derive a novel, prognostic 158 genes signature common to BRCA and STAD Claudin^low^ tumours. Finally, we identify splicing factors associated to high NF-YAr, validating RBFOX2 as promoting expression of NF-YAl. These data bring three relevant results: (i) the definition and clinical implications of NF-YAr and the 158 genes signature in Claudin^low^ tumours; (ii) genetic evidence of 28 amino acids in NF-YAl with EMT-promoting capacity; (iii) the definition of selected splicing factors associated to NF-YA isoforms.

## Introduction

Breast cancer (BRCA), the most common malignancy worldwide in women, is curable in 70% of cases, especially when identified at an early, non-metastatic stage [1]. It has been molecularly classified into different subtypes based on histopathological features and gene expression patterns. The use of Prediction Analysis of Microarray 50 -PAM50- was developed to identify four subtypes -Luminal A, Luminal B, HER2 + and Basal-like- exhibiting specific patterns of gene expression [2]. In parallel, the use of immunohistochemical (IHC) techniques concurs in the identification of the molecular subtypes. Unfortunately, neither PAM50 nor IHC-based techniques are absolutely accurate for clinical diagnosis, with numerous samples escaping from categorization. Recently, a discrete Claudin^low^ subtype was identified by bioinformatic means [3], characterized by low expression of critical cell adhesion molecules, including Claudin 3, 4 and 7, Occludin and E-cadherin, previously noticed in selected BRCA samples [4]. In general, commonalities are shared between Claudin^low^ and Basal-like tumours, among which mesenchymal and stem cell features [3].

Regulation of gene expression is at the heart of all developmental processes, including deviation from physiological patterns, such as cellular transformation and formation of tumours. The initial stage of gene expression is driven by the binding of sequence-specific Transcription Factors -TFs- to discrete promoter and enhancer elements. By analysing the structure of promoters driving expression of genes specifically overexpressed in cancer, many studies reported the overrepresentation of a specific element, the CCAAT box [5–10].

The TF regulating CCAAT box is the trimeric NF-Y, proven to be crucial for expression of many genes. It consists of three subunits, the Histone Fold Domain -HFD- NF-YB/NF-YC and the sequence-specific NF-YA. Several studies reported the overexpression of NF-Y subunits in different human cancers [11–18]. Typically, this came from analysing circuitries of TFs mediating transformation and NF-Y subunits were queried for increased expression. Most reports pointed to NF-YA as the overexpressed subunit in cancer, including our systematic analysis in TCGA and other datasets of breast, lung, liver, head and neck, prostate and stomach carcinomas [19–25].

A further level of complexity is brought by alternative splicing -AS- whose alterations have been shown to play a crucial role in the development of different types of tumours [26]. AS is governed by sets of proteins that guide either the inclusion of exons in the mature mRNA or their excision from the primary RNA transcript [27]. The NF-YA and NF-YC subunits are involved in AS: specifically, two major isoforms of NF-YA exist, NF-YAs (NF-YA short) and NF-YAl (NF-YA long), the latter comprising 28/29 amino acids within the large Q-rich Trans-Activation Domain (TAD), coded by exon-3. Both share the parts required for heterotrimerization and CCAAT-binding. We found that higher levels of NF-YAl correlates to a mesenchymal phenotype in BRCA, LUAD, HNSCC and STAD and a high ratio between NF-YAl and NF-YAs is predictive of a poor clinical outcome [19–21, 23, 25]. In particular, BRCA tumours and cell lines with a Claudin^low^ phenotype express high NF-YAl levels. Recently, it has been shown that these tumours are separate from the BRCA Basal-like subtype, representing a fifth individual molecular subtype [28]. In general, Claudin^low^ tumours are not restricted to BRCA, since similar subtypes have been classified in Bladder (BLCA) and Stomach Adenocarcinomas, based on gene expression signatures resembling Claudin^low^ tumours [29, 30].

We performed wet and in silico experiments to explore the role of the NF-YA isoforms, initially in BRCA, then extending our findings to gastric cancers. We formalized the importance of the NF-YAr concept and derived a Claudin^low^ signature common to BRCA and STAD. Finally, we explored the role of splicing factors mediating expression of the NF-YAl isoform.

## Results

### Deletion of NF-YA exon-3 in Claudin^low^ BRCA lines

Claudin^low^ breast cancer cell lines mostly express NF-YAl [19]. To understand the role of the isoform in breast cancer, we genetically ablated exon-3, coding for the 28 extra amino acids of NF-YAl, in two different Claudin^low^ cell lines, SUM159PT and BT549. We used the same strategy employed in murine cells [31], based on four guides flanking exon-3 and single strand-cutting Cas9-nickase (Cas9n), thus minimizing off-target effects (Supplementary Fig. S1A). After transfections, individual clones were isolated, expanded and genomic DNA screened by PCR, as shown in Supplementary Fig. S1B. We selected two clones with correct ablation in homozygosity for both cell lines and three random control clones without deletion (Supplementary Fig. S1C). The PCRs of clones #266 and #321 of SUM159PT, #12 and #242 of BT549, do not show the expected bands for the A amplicon, present only in parental cells and control clones, and for the B amplicon, shorter in edited clones respect to the controls (Supplementary Fig. S1C). Sequencing confirms deletion of exon-3, with somewhat different ends in the four YAl-KO clones (Supplementary Fig. S1D). We then checked isoform-specific mRNA expression: we did not score signals of NF-YA long transcript in edited clones (Supplementary Fig. S1E). Finally, analysis of protein levels by western blot confirmed exclusive expression of the NF-YAs in edited cells at comparable levels. As controls, NF-YB and NF-YC protein levels were assessed and found unchanged (Supplementary Fig. S1F).

### NF-YAl sustains in vitro migration capacities of breast Claudin^low^ cells

SUM159PT and BT549 YAl-KO clones are stable upon repeated cycles of freezing/thawing and their morphology looks apparently similar to the parental cells and control clones (Supplementary Fig. S2A). We assessed the area occupied by cells and reported no difference in size (Supplementary Fig. S2B), nor in growth curves (Supplementary Fig. S2C). We also checked clonal expansion by clone formation assay: compared to controls, the number of colonies formed in YAl-KO cells was not statistically different. (Supplementary Fig. S2D).

**Fig. 1:**
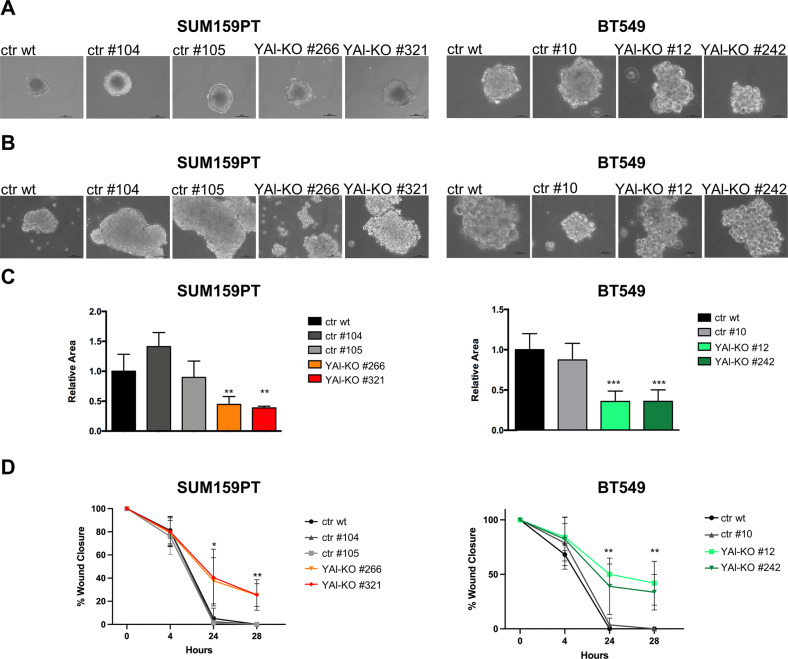
In vitro characterization of NF-YAl-KO edited clones and controls. **A** Transmitted light images of SUM159PT and BT549 spheroids formation assay performed in complete medium. **B** Transmitted light images of SUM159PT and BT549 disaggregated and replated spheroids grown in complete medium. **C** Evaluation of invasion ability with transwell assay of SUM159PT and BT549; each bar represents mean value and error bars the SD of at least two independent experiments performed (*p < 0.05, **p < 0.01, ***p < 0.001). **D** Migration evaluation ability through wound healing assay of SUM159PT and BT549; each point represents mean value and error bars the SD of three independent experiments performed (*p < 0.05, **p < 0.01, ***p < 0.001).

We then employed the spheroid formation assay, a three-dimensional system, as a measure of cell-cell contacts and extracellular matrix formation. YAl-KO clones of SUM159PT and BT549 formed aggregates, whose morphology look looser and less compact than spheres formed by controls, and they did show many single, detached cells in the plates (Fig. 1A). This phenomenon was confirmed by a disaggregation-replating procedure, as shown in Fig. 1B. The looser aspect of YAl-KO clones suggested us to investigate their motility and invasion abilities, typical of Claudin^low^ cells. Transwell assays showed that YAl-KO clones significantly lost the capacity to migrate (Fig. 1C), and wound healing migration assays confirmed that YAl-KO clones fill the wound more slowly than controls (Fig. 1D). Altogether, these data indicate that expression of NF-YAs in Claudin^low^ cells guarantees basal growth features, but NF-YAl is involved in supporting invasion and migration. Of note, these features were shared by independent clones of two different Claudin^low^ cell lines.

### Ablation of NF-YA exon-3 impairs in vivo migration of breast Claudin^low^ cells

**Fig. 2:**
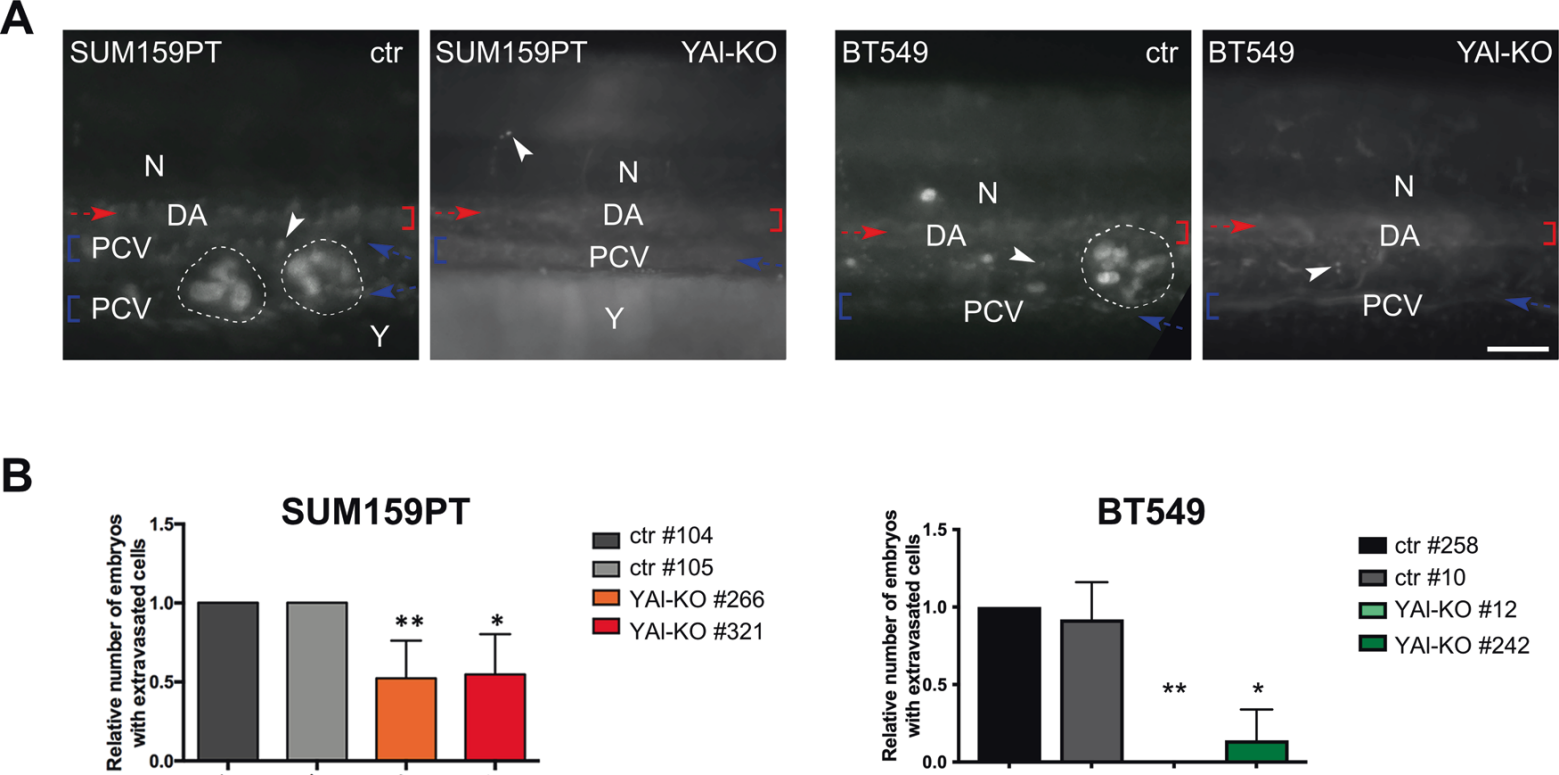
In vivo characterization of NF-YAl-KO edited clones and controls. **A** SUM159PT and BT549 cancer ctr and YAl-KO were fluorescently labelled with Hoechst 33342 solution and directly injected into the Perivitelline Space (PVS) of 48 h post-fertilization (hpf) zebrafish larvae. Total number (*n*) of circulating-tumour-cells larvae analysed: SUM159PT ctr, *n* = 98; SUM159PT YAl-KO, *n* = 112; BT549 ctr, *n* = 105; BT549 YAl-KO, *n* = 68. All images are taken 24 h post-injection (hpi) and show the caudal haematopoietic tissue (CHT) tail region. The red and blue brackets indicate the DA and PCV, respectively, while the red and blue arrows show the blood flow in the two vessels. The white-dotted perimeters indicate the metastases (in the first image on the left of the panel the PCV is split in 2 branches because of the metastatic clusters). The arrowheads point out single circulating tumour cells. N notochord, DA dorsal aorta, PCV posterior cardinal vein, Y yolk. Scale bar: 100 µm. Anterior left, dorsal top. **B** Evaluation of migration ability through 48 hpf zebrafish embryos injection. Each bar represents mean value % of embryos that 24 hpi showed metastasis; error bars represent the SD of at least two independent experiments performed (*p < 0.05, **p < 0.01).

Claudin^low^ breast cancer cells are highly invasive in vitro and metastasize in experimental and murine models in vivo [32]. To test the in vivo invasion/metastasis potential of the YAl-KO clones, we employed zebrafish, a widely used biosystem which allows to monitor these features. SUM159PT and BT549 YAl-KO clones and control cells were microinjected into the perivitelline space of 48 h post-fertilization (hpf) embryos and analysed 24 h post-injection (hpi), using an inverted fluorescent microscope. The spreading of fluorescently labelled extravasated cancer cells could be seen throughout the body of the fish, but predominantly in the area of the caudal haematopoietic tissue (CHT), located in the tail region (Fig. 2A). As expected, cells from SUM159PT and BT549 control clones clearly metastasize at 24 hpi, and the extent of metastasis was readily apparent, with large clusters of cells. The same results were obtained at 48 hpi (data not shown). Instead, SUM159PT YAl-KO clones showed less invasion potential and the BT549 ones hardly any (Fig. 2B). Moreover, they rarely showed extravasated cells in the body of the fish (Fig. 2). These correlations were consistent within the cohorts of fish used for each individual experiment. These data agree with in vitro invasion assays, further suggesting that NF-YAl is specifically involved in the metastatic process.

### NF-YAl regulates distinct pathways, including EMT

**Fig. 3:**
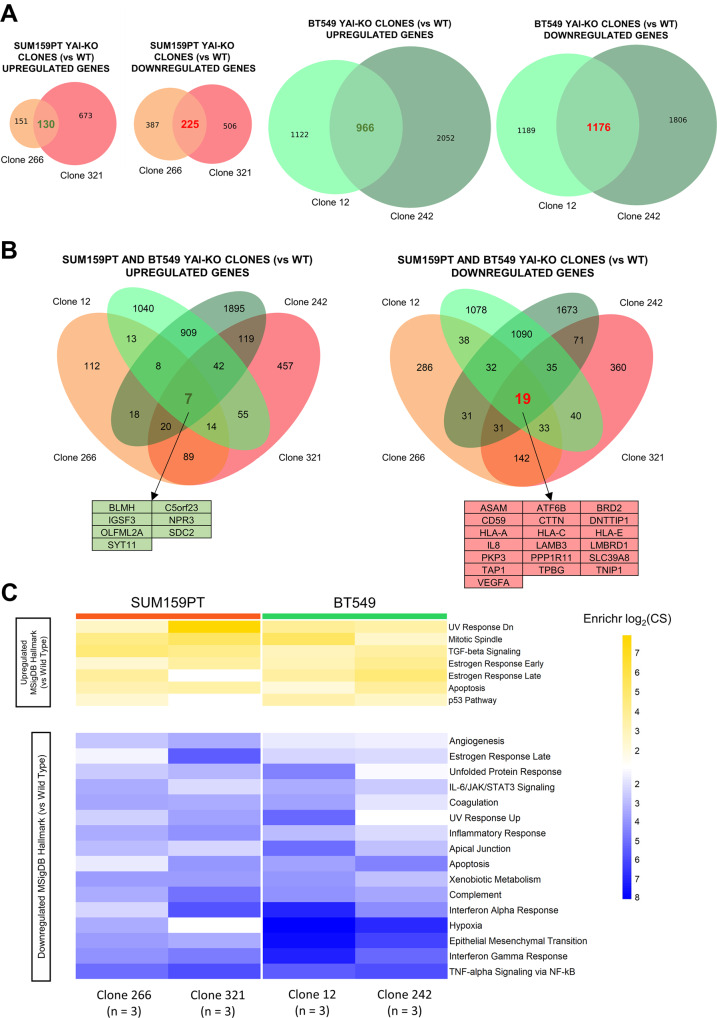
SUM159PT and BT549 NF-YAl-KO clones differential expression and functional analysis. **A** Venn diagrams showing the overlap of differentially expressed genes (DEGs) in SUM159PT (Left Panel) and BT549 (Right Panel) YAl-KO clones, compared to the respective wt cell-lines. **B** Venn diagrams representing the number of shared DEGs between all four YAl-KO clones, compared to wt cell lines. In the Left Panel we included upregulated genes, in the Right Panel downregulated ones. Tables at the bottom detail the genes up- or downregulated in all four YAl-KO clones. **C** Heatmap depicting enriched MSigDB Hallmark gene sets within the DEGs (vs wt cell lines) of each deleted clone. Upregulated and downregulated gene sets are displayed in yellow and blue, respectively. Intensity of the colour = -log_2_(Enrichr Combined Score). Only gene sets with -log_2_(CS) > 1 in all four DEG lists were included in the analysis.

To identify the deregulated pathways involved in the altered migratory capacities of YAl-KO clones, we performed RNA-seq of SUM159PT and BT549 YAl-KO clones and wt controls, defining differentially expressed genes, DEGs (Fig. 3A; listed in Supplementary Table S1). We observed that the genes collectively up- or down-regulated were relatively few in BT549 and more numerous in SUM159PT. This discrepancy is most likely due to the respective identities and to specific features of individual clones (Fig. 3B). Yet, extrapolation of the deregulated pathways led to the identification of common terms such as *EMT process*, *inflammatory signalling* (IFN), and *hypoxia* as collectively downregulated in YAl-KO clones of both cell lines (Fig. 3C). *EMT process* is expected, as its downregulation is consistent with the loss of migratory capacity of the YAl-KO clones. Intriguingly, *inflammatory* and *interferon signalling* genes, typically considered as tumour suppressive, are also downregulated, in keeping with such genes found overexpressed in breast Claudin^low^ tumours and correlating with worst prognosis [33, 34]. Overall, these terms suggest a less aggressive phenotype of the YAl-KO clones. Concerning upregulated terms, we found *p53* and *Estrogen Response pathways*, which seem counterintuitive, since Claudin^low^ belongs to the “triple negative” tumours, featuring loss of ERalpha expression and mutation of p53. However, the switch to NF-YAs could impact on the expression of genes previously classified as ER-responsive lacking EREs, hence independent from ER activities, characterized by the presence of E2F1, NF-Y and NRF1 motifs in their promoters [35]. As for p53, BT549 cells carry R249S, a “conformational” mutation *a-la* R175H: such mutants mostly behave like those impacting on DNA-binding (R248 and R273), that is, as Dominant Negatives, rather than Loss-of Function: for example, R249S fails to repress, and rather activates certain targets [36]: the differential regulation in the context of the two isoforms is an interesting issue worthy of further investigation.

### A NF-YAr signature characterizes Claudin^low^ breast cancers

**Fig. 4:**
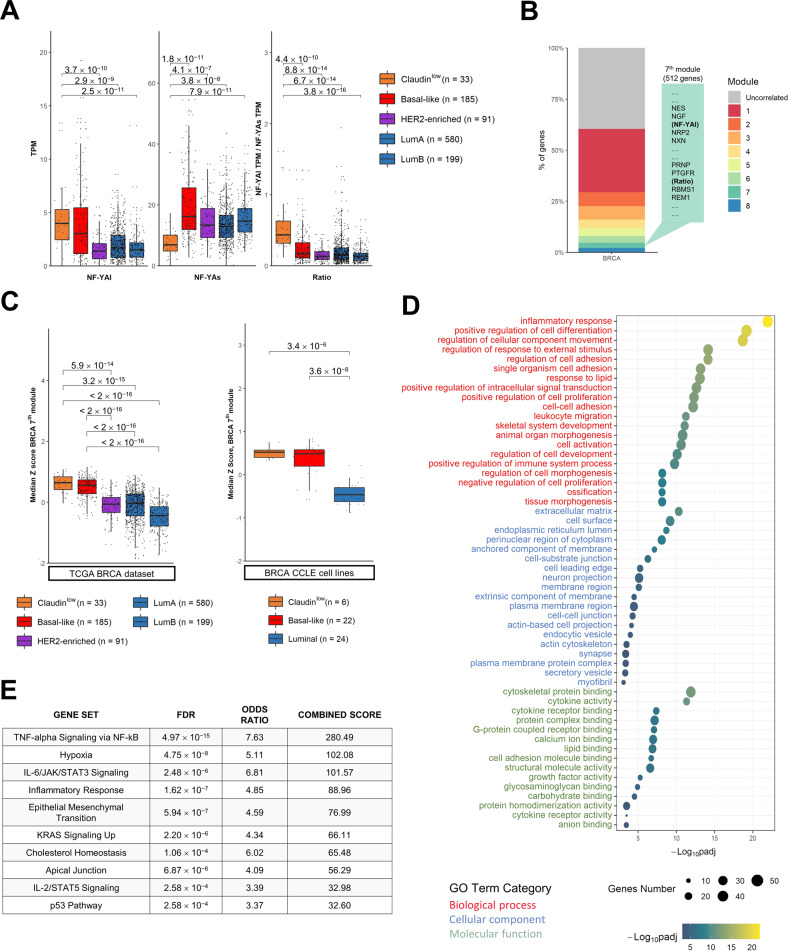
BRCA NF-YAl/NF-YAr WGCNA gene module characterization. **A** Expression levels of the NF-YA isoforms and NF-YAr in the TCGA-BRCA dataset, measured in TPMs. Samples are divided according to the new DeepCC classification. **B** graphical representation of the 8 gene modules determined by weighted gene co-expression network analysis, with the associated proportion of total genes. The module depicted in grey gathers uncorrelated genes. **C** Box plots of the median Z scores calculated for each sample across genes of the BRCA WGCNA 7^th^ gene module, in the TCGA-BRCA dataset (Left Panel) with samples divided according to the DeepCC classification, and in CCLE (Right Panel), where cell lines are separated following the classifications from Prat et al. [65] and Dai et al. [66]. **D** KOBAS-generated list of GO terms enriched in BRCA WGCNA 7^th^ module. Terms are ranked according to -log_10_(*q* value) and divided within the three main GO categories. Dot sizes represent the genes shared by the module and each GO term. **E** Most enriched MSigDB Hallmark gene sets in BRCA WGCNA 7^th^ module, as calculated by the Enrichr website. *p* values in (A) and (C) box plots are calculated using the Wilcoxon rank-sum test.

We previously reported on altered splicing of NF-YA isoforms in BRCA of TCGA and independent GEO datasets [19]. Traditionally, four subtypes have been described for BRCA, but more recently, the TCGA dataset has been re-classified into 5 distinct subtypes using the PAM50 signature [28]. Before proceeding with further analysis, we used the DeepCC machine learning method to classify all samples, including 28 Normal-like and 109 previously termed as Unclassified, using as training set the already classified tumours [22]. We checked the distribution of the new categorization (Supplementary Fig. S3A): only a small number of samples (*n* = 12) remained unclassified, with the majority being in the Luminal subtype (Supplementary Fig. S3B). Expression of NF-YA isoforms and their ratio (NF-YAr = NF-YAl/NF-YAs) was previously assessed according to the four subtypes classification [19]: we repeated this analysis with the complete classification, as in Fig. S3. This confirmed that the Claudin^low^ subtype shows highest expression of NF-YAl and lowest of NF-YAs, making NF-YAr higher in this subset (Fig. 4A). As expected, Basal-like tumours also show a similar trend.

Next, we extracted NF-YAr-associated gene signatures from RNA-seq datasets of all TCGA specimens. We replaced gene-level NF-YA expression with NF-YAl and NF-YAs at isoform-level, and we treated NF-YAr as a “normal” gene. Exploiting WGCNA (Weighted Gene Co-expression Network Analysis) and considering only tumour samples, 8 gene clusters emerged (Fig. 4B). NF-YAr and high NF-YAl expression grouped together in Module 7, suggesting that the ratio is influenced prevalently by the amount of NF-YAl (Fig. 4B). Module 7 is composed of 512 genes (Supplementary Table S2): plotting their expression according to the subtypes, we observed expression mostly in Claudin^low^ and in Basal-like (Fig.4C, Left Panel). Supplementary Fig. S4 shows a heatmap of gene expression according to the 8 modules and divided according to subtypes. Analysing the NF-YAr-driven 7^th^ Module in BRCA cell lines expression data from CCLE, a significant correlation with Claudin^low^ and, to a lesser extent, Basal-like also emerged (Fig. 4C, Right Panel). Functional analysis of the NF-YAr signature revealed categories belonging to differentiation, specifically of tissues of mesenchymal origin; terms associated to cell adhesion and extracellular matrix emerged as the most deregulated GO categories (Fig. 4D). We also investigated the enrichment in MSigDB Hallmarks: inflammatory pathways and epithelial to mesenchymal transition were characterized as the most enriched sets (Fig. 4E). These data are in line both with the genetic and gene expression data of edited Claudin^low^ cell line shown above.

### A NF-YAr 158 genes signature common to Claudin^low^ BRCA and STAD tumours

**Fig. 5:**
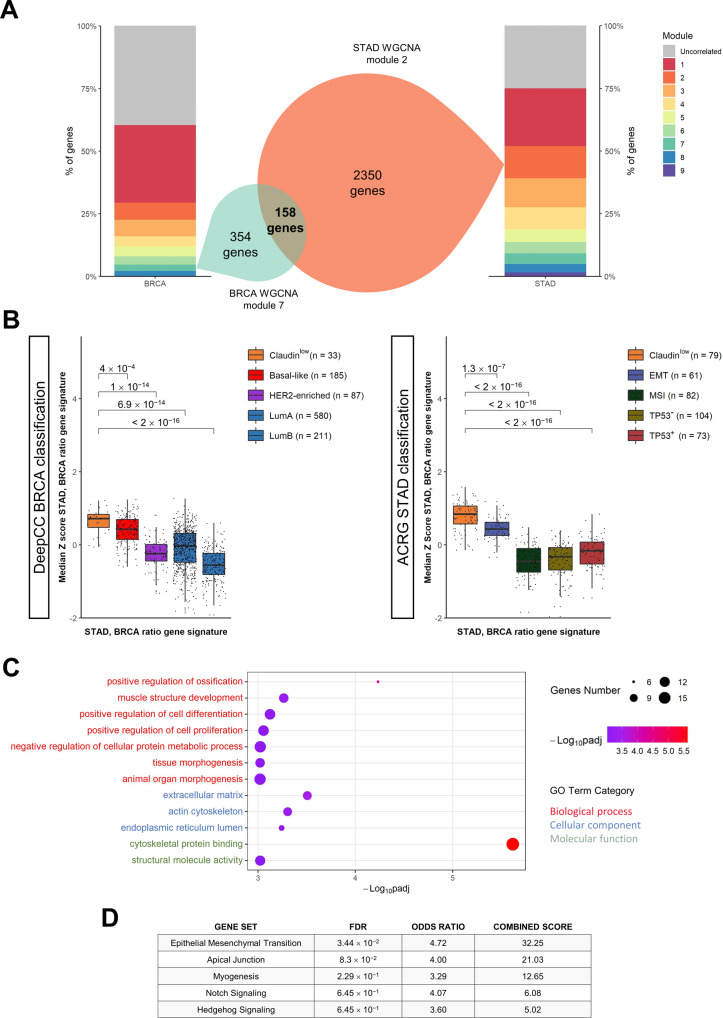
BRCA-STAD NF-YAr Signature definition and characterization. **A** Overlap between BRCA WGCNA 7^th^ module and STAD 2^nd^ module, including NF-YAl and NF-YAr. The stacked bar plots at the borders represent the percent of total genes belonging to each module in the two cohorts, and the grey module includes uncorrelated genes. **B** Box plots of the median Z scores calculated for each sample across the genes of BRCA-STAD NF-YAr gene signature, in the TCGA-BRCA dataset (Left Panel) and in TCGA-STAD dataset (Right Panel). Samples are distributed according to the respective DeepCC classifications. p values are calculated using the Wilcoxon rank-sum test. **C** GO terms enriched in BRCA-STAD NF-YAr gene signature, as computed by KOBAS. Terms are ranked according to -log_10_(q value) and divided within the three main GO categories. Dot sizes represent the number of genes shared by the module and each GO term. **D** Most enriched MSigDB Hallmark gene sets in BRCA-STAD NF-YAr gene signature, as calculated by Enrichr.

We recently reported on higher levels of NF-YAl in Claudin^low^ samples of gastric cancer [25]. We thus started to investigate TCGA STAD samples to define a NF-YAr signature that better marks Claudin^low^ tumours, by integrating co-expression network analysis. With the same pipeline described above, we extracted the genes clustering with NF-YAl and NF-YAr expression in gastric samples. Both fell in the same cluster in gastric cancer with 2508 genes. To restrict the NF-YAr signature, we extrapolated the genes commonly correlating with NF-YAr in both breast and gastric cancer, retrieving 158 genes (Fig. 5A and listed in Supplementary Table S2). We tested this signature across the different subtypes: Fig. 5B shows it mainly characterizes Claudin^low^ TCGA tumours of BRCA (Supplementary Fig. S3) and in STAD, the latter derived by using subtypes according to the ACRG classification, added of Claudin^low^ [25]. Functional characterization of the 158 genes indicates involvement in mesodermal related pathways and Gene Ontology indicates differentiation and muscle related categories (Fig. 5C); MSigDB Hallmark gene sets enrichment analysis further confirmed the GO results, highlighting pathways related to EMT and Myogenesis (Fig. 5D).

In conclusion, we identify a 158 genes signature defining Claudin^low^ tumours common to BRCA and STAD, correlating with high NF-YAr.

### Prognostic value of NF-YAr and the 158 gene signature

**Fig. 6:**
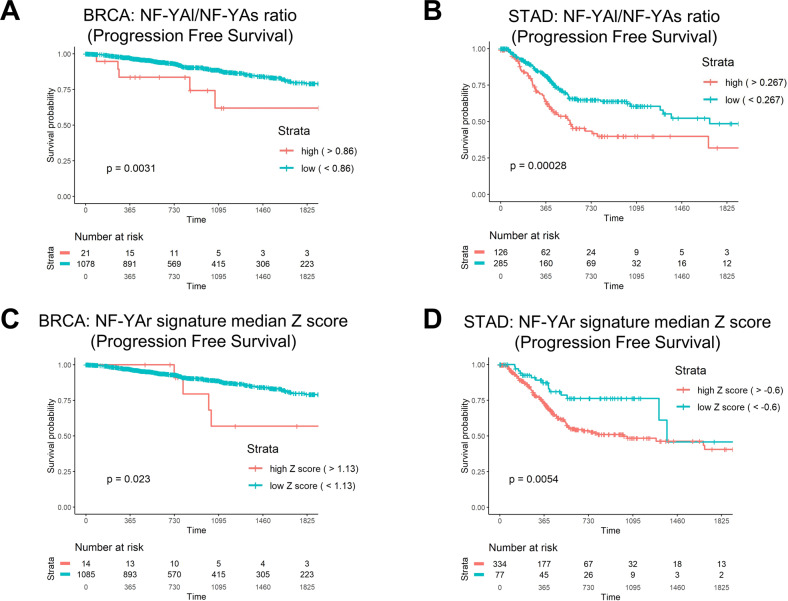
Clinical analysis of NF-YAr in TCGA BRCA and STAD cohorts. Progression-Free-Interval curves of survival probability, with stratification according to the Cutoff Finder-determined threshold for NF-YAr (0.86) in TCGA BRCA patients (**A**), for NF-YAr in STAD (**B**) threshold = 0.27, for the NF-YAr signature median Z score in BRCA (**C**) threshold = 1.13, and for the NF-YAr signature median Z score in STAD (**D**) threshold = −0.60. *p* values are calculated using the log-rank test.

We assessed the prognostic value of NF-YAr in the TCGA BRCA and STAD cohorts, independently from the subtype classification of samples. First, through the Cutoff Finder web tool, we found that the optimal NF-YAr value for dichotomization of Progression Free Survival -PFS- of BRCA samples is 0.86. The NF-YAr-directed stratification of BRCA samples shows an unbalanced distribution, yet survival analysis confirms that partition of samples with this cut-off predicts the survival outcome (Fig. 6A). Using the same approach, the cut-off in STAD was set to 0.27, predicting a threshold above which STAD patients show an unfavourable outcome (Fig. 6B). The same exercise was repeated with the 158 Claudin^low^ signature, yielding similar results in BRCA (Fig. 6C) and STAD (Fig. 6D). In summary, despite a numeric disproportion of Claudin^low^ samples –STAD ≫ BRCA- both NF-YAr and the 158 signature are predictive of a worst prognosis.

### High NF-YAr correlates with EMT cancer cells in scRNA-seq BRCA and STAD

**Fig. 7:**
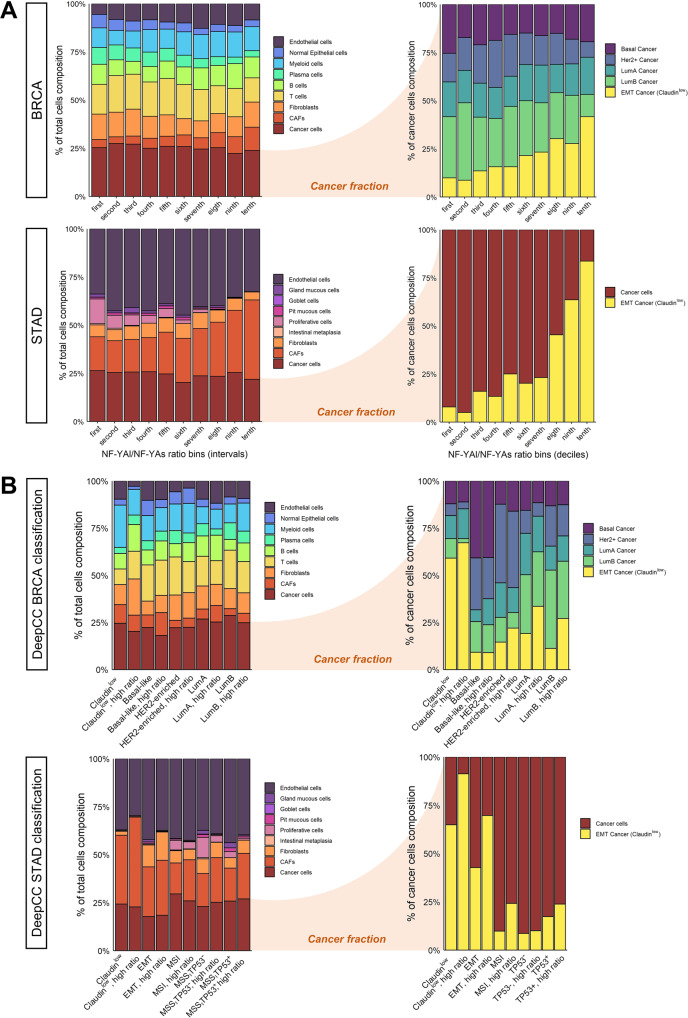
Cell type deconvolution of TCGA BRCA and STAD samples. **A** Proportion of cells annotated as each cell type, as predicted by the SDCD deconvolution package, for TCGA BRCA and STAD samples: in the Left Panel all cell types constituting the tumour microenvironment are considered, whereas on the Right only cancer cells are evaluated. TCGA samples are divided according to NF-YAr deciles, and the average cell type proportions among samples comprising each decile are plotted. **B** Same as (A), except that TCGA BRCA and STAD samples are partitioned according to their respective DeepCC classifications.

Bulk RNA-seq experiments have the limit of measuring gene expression of cell types within a tumour, including cancer cells. To discriminate correlations between NF-YAr and specific cell types, we exploited single cells experiments of breast [37] and gastric cancers [38]; we used the respective signatures to deconvolute the cell population composition of TCGA breast and gastric samples. In Fig. 7A, we ranked samples of the two cohorts, divided in deciles, according to NF-YAr, and we plotted average cell populations as predicted by the SCDC deconvolution tool. Both datasets show a slight increase in cancer-associated fibroblasts (CAFs) in samples with high NF-YAr values. Focusing on the predicted cancer cell population, we predict the composition of these cells in terms of cancer cells subpopulations: we observed a very robust correlation between the proportion of EMT cancer cells and NF-YAr (Fig. 7A, right panel). In addition, the BRCA Luminal population anticorrelated with NF-YAr values. Recently, the Claudin^low^ subtype emerged as separated, found primarily in Basal-like tumours and less in other subtypes [28]. We tested our NF-YAr predictor also inside the standard subtype framework: in Fig. 7B, we observed that within each subtype, the subset of samples with high NF-YAr correlated with a more expanded population of EMT cancer cells in breast and gastric, except for Basal-like. In summary, deconvolution analysis according to scRNA-seq data supports the hypothesis that high NF-YAr is predictive of cancer cells with an EMT phenotype.

### RBFOX2 promotes NF-YA exon-3 inclusion in Claudin^low^ tumours

**Fig. 8:**
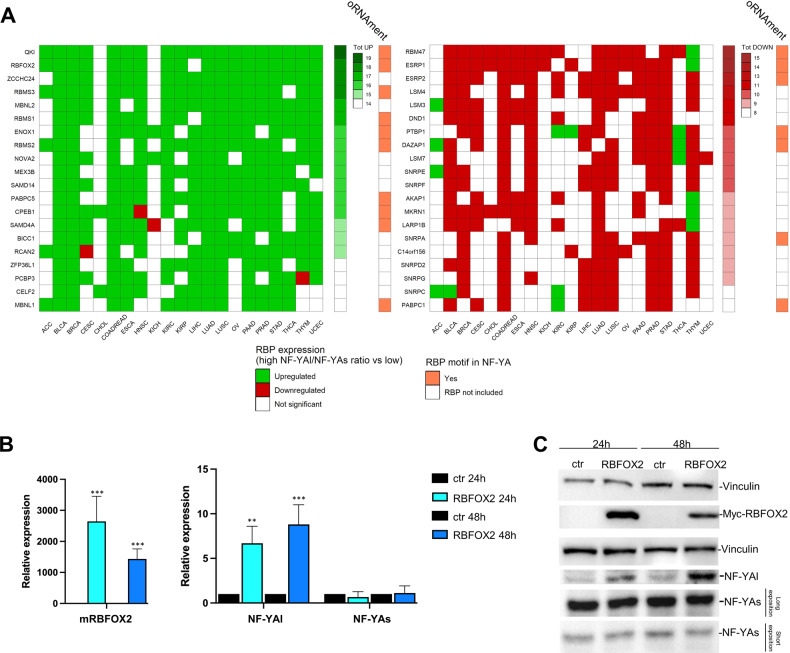
RBFOX2 correlation with NF-YA exon-3 inclusion. **A** Heatmaps depicting variation of RBPs expression between high NF-YAr samples (fourth quartile) and all others, in 21 TCGA epithelial cancer cohorts. Green squares represent significant overexpression (*p* < 0.05) in high NF-YAr samples, as tested by Wilcoxon rank-sum test, and red squares are associated to lower expression values. Left Panel: RBPs are ranked according to the number of cohorts in which they are significantly overexpressed (“Tot UP” column). Right Panel: RBPs are ranked according to the number of cohorts in which they have a significantly lower expression (“Tot DOWN” column). Only the top 20 RBPs are shown in both panels. The oRNAment column illustrates the presence of each RBP binding site motif within the NF-YA locus. **B** Gene expression analysis of mouse RBFOX2, NF-YA long and NF-YA short levels in T47D luminal cells transfected with plasmid carrying mRBFOX2 gene or with an empty plasmid. Each bar represents mean value and error bars the SD of at least two independent experiments performed (*p < 0.05, **p < 0.01, ***p < 0.001). **C** Western Blot analysis of MYC-RBFOX2, NF-YA short and NF-YA long in T47D luminal cells transfected with plasmid carrying mRBFOX2 gene or with the empty control.

The above data showing that altered splicing of NF-YA isoforms has an impact on BRCA and STAD tumours begs the question as to which splicing factor is responsible for this behaviour. First, we interrogated the TCGA repository by analysing all the epithelial cancers cohorts: we divided tumour samples for each type based on quartiles of NF-YAr, defining two groups: high NF-YAr samples associated to the fourth quartile, and low NF-YAr group with all other samples. Then, we compared expression of the RNA binding protein (RBPs) genes included in the RBP database [39] in the two groups. Fig. 8A shows the RBPs that significantly correlated (on the left) and anticorrelated (on the right) in expression with high levels of NF-YAr. The classification was performed ordering RBPs according to the number of cancer cohorts in which we found a significant correlation, trying to predict the candidate RBPs responsible for NF-YA isoforms switching. On the top of the correlating list, we found QKI and RBFOX2, while RBM47 and ESRP1 resulted as the most anticorrelating (Fig. 8A). In parallel, we searched for RBPs motifs in the NF-YA exon-3 flanking introns through the oRNAment tool [40]: we confirmed the presence of direct sites of RBFOX2 and QKI in NF-YA transcripts as indicated in Fig. 8A. We checked the expression of the top 20 correlating and anticorrelating RBPs across BRCA and STAD subtypes: the heatmaps of Supplementary Fig. S5 shows high expression of most correlating RBPs in Claudin^low^ samples, and low in LuminalA/B. The reverse is observed for anticorrelating RBPs. Finally, we analysed CCLE BRCA lines: high NF-YAr-related RBPs -MBNL1/1, QKI and RBFOX2- are expressed predominantly in Claudin^low^ lines; CELF2, instead, appears to be expressed almost exclusively in specific Basal-likes. Instead, the anticorrelating RBPs ESRP1 and RBM47 are specifically lowly expressed in Claudin^low^ lines and show high expression in Luminal cells (Supplementary Fig. S6): in such lines, we previously detected almost exclusive expression of NF-YAs [19].

To substantiate these findings, we analysed available RNA-seq experiments in which correlated and anticorrelated RBPs were functionally inactivated, or overexpressed, for expression of NF-YA isoforms. Upon alteration of levels of these RBPs, we scored a significant impact on isoforms expression, namely increase of NF-YAs in MBNL1/2, QKI and -markedly- RBFOX2 inactivation, and increase of NF-YAl in RBM47 and ESRP1-2 inactivation (Supplementary Fig. S7). To further validate our predictions, we overexpressed RBFOX2 in Luminal breast cells, chosen after analysis of the RBPs expression patterns across CCLE breast cell lines, confirming the same trend observed for tumour subtypes (Supplementary Fig. S6). We overexpressed RBFOX2 in the luminal cell line T47D, with prevalent expression of NF-YAs: as shown in Fig. 8B we observe maximum expression of RBFOX2 at 24 h, and after 48 h we observed an increase of the NF-YAl transcript. We confirmed this also at the protein level, with the peak of NF-YAl expression at 48 h post-transfection (Fig. 8C). In conclusion, bioinformatic and wet biology experiments suggest that two RBPs -RBFOX2 and QKI- are involved in controlling the levels of NF-YA isoforms, specifically increasing NF-YAl.

## Discussion

Our work sheds light on the importance of the individual NF-YA isoforms for the biology of BRCA and STAD. The role of NF-YAl was studied in vitro and in vivo by gene editing; bioinformatic analysis, including deconvolution of scRNA-seq data, led to the identification of a NF-YAr signature shared by Claudin^low^ BRCA and STAD. Finally, we provide evidence of a role of selected AS proteins in influencing NF-YA isoforms levels.

### NF-YA isoforms

The ubiquitous presence of NF-Y-binding activity in all cell lines, of CCAAT sites in promoters of disparate genes and the results from YAl-KO experiments signalling an essential role of NF-YA in early embryogenesis, all contributed to the conclusion that NF-Y, including NF-YA, is not relevant for cellular transformation. This view changed when it was realised that NF-YA is absent in some post-mitotic cells, it is associated to cell proliferation in vivo and that the targeted genes are enriched in growth-promoting categories, namely cell-cycle progression and metabolism. Not surprisingly, functional removal of NF-YA -by siRNA or shRNA- leads to impairment -or lack- of cell growth [41]; conversely, overexpression promotes different aspects of cell proliferation and tumorigenesis [24, 42–45]. Overexpression of TF genes can have widespread direct -and indirect- transcriptional consequences, and this is most likely true for NF-YA, whose protein expression levels are tightly controlled [46]. Indeed, stable, complete ablation of NF-YA in cell lines has not been reported so far, being likely lethal for growing cells.

We used genetic means to ablate exon-3, generating Claudin^low^ cells lines expressing physiological levels of NF-YAs instead of NF-YAl. Basic features -morphology, growth rates- of the YAl-KO clones are apparently unaffected, at least in the conditions tested. We reported similar findings with mouse myoblasts C2C12, substituting NF-YAl with NF-YAs by genome editing [31]. The two Claudin^low^ lines used here are different in morphology, growth rates and expression profiles, but they share commonalities, such as high migratory capacities, including to metastasize in vivo: in both lines, these features are substantially diminished -or lost- in YAl-KO clones expressing NF-YAs.

Over the last few years, several groups reported that NF-YA is overexpressed in cancers, specifically epithelial ones [19] and that the roles of the two major isoforms might be fundamentally different. In reports on endometrial carcinoma (EC), two studies reported a role of NF-YAs in aggressive ALDHA1^+^ tumours [47] and a direct correlation with EMT markers in high-grade EC [18]. A recent report delineates a complex interplay between the two isoforms in prostate cancer (PCa): on the one hand, NF-YAs levels are higher in LumB, the subtype with the worst clinical outcome, and overexpression increases spheroids and xenografts tumours [24]. However, a significant increase in the NF-YAl/NF-YAs ratio distinguishes circulating tumour cells from cells within metastasis: this is consistent with increased pro-migratory functions observed in NF-YAl-overexpressing PCa lines. This paints a scenario in which NF-YAr is modulated according to different stages of tumour progression: first NF-YAs (increased proliferation), then NF-YAl (detachment and migration to distant sites), finally NF-YAs again within metastasis (attachment/homing and growth?). In BRCA and STAD, instead, we report that locally aggressive Claudin^low^ tumours, irrespective of their metastatic status, have already high NF-YAr, which is a clinically impactful concept. Finally, an additional NF-YA isoform -NF-YAx, devoid of both exon-3 and exon-5- is expressed in neuroblastoma (NB) [48]. Overexpression of NF-YAx activates key genes -Nestin, SOX2, Nanog- that lead to selection of NB cancer stem cells. Note that all isoforms, including NF-YAx, share the conserved HAP2 domain responsible for heterotrimerization and efficient and selective DNA-binding, thus all CCAAT box-containing genes could be targeted and activated. These findings are intriguing, since this shorter version lacks large parts of the TAD, including stretches we recently identified as extremely conserved in evolution [49], one of which proven to be functionally important [50]. In fact, NF-YAx loses the capacity to interact with Sp1/3, well known NF-Y partners in activation, thus it is expected to have negative effects on expression of some genes. We have been unable to detect relevant levels of NF-YAx in the epithelial tumours we analysed, so this isoform might be very specific for neuronal malignancies.

### NF-YAr

Our bioinformatic analyses expand previous concepts and provide new insights into the association between the ratio of NF-YA isoforms and epithelial tumours, particularly Claudin^low^. First, we confirm previous reports suggesting that Claudin^low^ tumours are a discrete subtype in BRCA and STAD, based on profiling analysis of the respective TCGA samples. Their number in BRCA is lower -some 3%- than in STAD, yet upon categorization of DEGs, they share common pathways and GO terms. Second, we previously reported on NF-YA overexpression in BRCA and STAD, and on high levels of NF-YAl in breast and gastric Claudin^low^ cell lines and tumours: we now define and quantify this concept of discrete values of NF-YAr. In turn, these are relevant to predict the clinical outcome of these tumours. Note that there is a difference in the values in the two tumours, but this might have to do with the higher number of Claudin^low^ tumours in STAD, generating more robust statistics. Third, using NF-YAr as feature, we derived a 158 genes signature common to BRCA and STAD. Note that previous Claudin^low^-based signatures, derived separately in BRCA and STAD, were substantially different. Thus, our data go along with the establishment of a unifying set of genes whose expression is commonly deregulated in these types of tumours, independently from the origin. This signature is enriched in EMT and mesenchymal terms in general, which is to be expected based on the previous and present analysis and on the genetic experiments and RNA-seq data presented in Figs. 1–3. In turn, these data directly impinge on the specific role of NF-YAl -and not NF-YAs- in these classes of genes. Fourth, by analysing scRNA-seq data to deconvolute BRCA and STAD cellular compositions present in TCGA samples, we found that there is a parallel increase of NF-YAr and the Cancer Associated Fibroblasts -CAF- population, but not that of normal fibroblasts, whose overall numbers are not affected. This is relevant, since CAFs are well known to play an important role in tumour expansion [51]. As for the population of cancer cells, which make up some 25% of both STAD and BRCA samples, the higher the NF-YAr, the more numerous are the cancer cells labelled as EMT. This effect is particularly striking in STAD samples. Even partitioning for tumour subtypes and high or low NF-YAr, the EMT phenotype follows well high ratios, further confirming that these features are intrinsically associated. We conclude that a substantial change in cellular behaviour is brought by exon-3 28 amino acids within the TAD. These results bring at least two avenues of future investigations: verification of the Claudin^low^ signature identified here in other epithelial tumours, in primis BLCA, and identification of the molecular mechanisms empowering these 28 amino acids with such specific transcriptional effects.

### RBPs in cancer

AS is taking a centre-stage in studies of expression profiling of tumours [52], and many studies increasingly focus on the roles of RNA-Binding Proteins involved in AS. A global change in expression of specific isoforms of hundreds of genes is routinely detected in tumours and, indeed, this goes along with changes in expression of individual RBP genes. We asked the obvious question, that is, which RBP affects differential splicing of the two NF-YA isoforms. By interrogating acknowledged members of the RBP database for correlation with high NF-YAr in TCGA tumour samples, some factors emerge; other RBPs, predictably, anti-correlate. Reassuringly, the top hits did show the presence of the respective sites within the exon-3 RNA splicing areas. We validated changes of NF-YA isoforms by analysing RNA-seq experiments previously performed after RBFOX2, CELF2, MBNL1-2 and QKI knock-down: all hits behaved as expected, namely inactivation of these genes entails a decrease of NF-YAl. RBFOX2 was further tested upon overexpression in a luminal breast line predominantly expressing NF-YAs, and indeed we detect an increase of NF-YAl at the mRNA and protein level. In fact, RBFOX2 is involved in alternative splicing in mesenchymal tissues and during the epithelial-mesenchymal transition process, which is important for cancer cell metastasis [53].

As to the RBPs that anti-correlate with high NF-YAr, thus promoting eviction of exon-3 and production of NF-YAs, RBM47 and ESRP1-2 are validated by RNA-seq data of knocked-down cells. Two further results are consistent with our data. Experiments on ESRP1/2 functional KO by shRNA interference reported altered NF-YAr ratios, with a substantial increase of NF-YAl and decrease of NF-YAs, which was prevalent in the cell line used [54]. In the context of cellular reprogramming of MEFs -expressing predominantly NF-YAl- to iPS -predominantly NF-YAs- Cieply et al. showed that inactivation of RBM47 leads to a shift of the NF-YA protein isoforms from NF-YAs to NF-YAl [55]. Epithelial specific alternative splicing is regulated by RBM47 that is also found inactive in some breast cancers and whose low expression in patients correlates with poor clinical outcome [56].

On the other hand, we were surprised not to find U2AF2 in the RBPs list emerging from our analysis. Conditional inactivation of U2AF1 in haematopoietic mouse cells led to failure of haematopoiesis due a defect in stem cell renewal, ultimately fatal [57]. NF-YA levels are decreased, along with other relevant TFs, such as PBX1, Meis1 and Runx2. Most importantly, U2AF1 inactivation by shRNA led to splicing alterations and decrease in NF-YA levels. Conversely, OE of NF-YA compensate for U2AF1-KO. These data are consistent with previous work showing a similar phenotype in mice in which NF-YA was conditionally KO in haematopoietic stem cells, mostly expressing NF-YAs [58]. The lack of U2AF1 in our lists might be due to tissue-specific effects since most datasets we analysed are from epithelial tumours. In general, it is possible that no single AS factor has a dominant role on NF-YA splicing, which might be coordinately controlled by several factors, partially in a tissue-preferred way.

In conclusion, we are left with a relatively small group of RBPs that are worth further genetic work: genome editing could shed light on the collective AS circuit in the cells we used. In addition, our data awaits the actual biochemical prove that these RBPs bind sequences in NF-YA exon-3 boundaries, in vitro and in vivo.

## Materials and methods

### Cell culture and transfection

Human breast cancer cells SUM159PT (ATCC) were cultured in DMEM/F12 (1:1) supplemented with 10% FBS (EuroClone), insulin 5 μg/ ml (Sigma-Aldrich), L-glutamine 1 mM 100 μg/ ml (EuroClone), Penicillin and Streptomycin 100 μg/ ml (EuroClone), Hydrocortisone 5 μg/ ml and HEPES 25 mM (EuroClone). BT549 (ATCC HTB-^122TM^) were grown in RPMI 1640 supplemented with 10% FBS, insulin 5 μg/ ml, L-glutamine 1 mM 100 μg/ ml, Penicillin and Streptomycin 100 μg/ ml. T47D cells (ATCC HTB-^122TM^) were cultured in DMEM/F12 (1:1) supplemented with 10% FBS, L-glutamine 1 mM 100 μg/ ml, Penicillin and Streptomycin 100 μg/ ml. All cell lines resulted negative for mycoplasma test. 4 × 10^5^ T47D cells were transfected with 4 μg of pIRESneo plasmids carrying mouse Myc-RBFOX2 or empty control with Lipofectamine_2000_.

### Genome editing

To target NF-YA exon-3, we used the same strategy employed in murine muscle cells [31], with four gRNAs (Supplementary Table S3) designed to target the introns flanking the human exon-3. 10^6^ SUM159PT and BT549 cells were transfected with 3 μg of the two gRNAs/nCas9 plasmids by electroporation (Nucleofector^®^ 2b Device). A total of 420 individuals clones for each line were isolated 72 h post-transfection and expanded. Clones were then screened for exon-3 deletion by genomic analysis by semi-quantitative PCR using the amplicons indicated in Supplementary Fig. S1B. Two homozygously deleted clones were identified for each line and thereafter sequenced to verify the deletion ends.

### RNA extraction, real-time PCR and RNA-seq

RNA samples were extracted with the Tri-Reagent protocol (Sigma-Aldrich). RNA samples were extracted, retrotranscribed and analysed as in murine cells [31]. RNA samples for RNA-seq were extracted from three independent biological replicates, treated with DNAse and checked for their quality by RNA ScreenTape Assay with TapeStation System. For BT549, mRNA was enriched using oligo(dT) beads, cDNA synthesis done using random hexamers and reverse transcriptase; a custom second-strand synthesis buffer (Illumina) was added with dNTPs, RNase H and Polymerase I to generate the second strand by nick-translation. After a round of purification, terminal repair, A-tailing, ligation of sequencing adapters, size selection and PCR enrichment, the cDNA libraries were sequenced. For SUM159PT, total RNA was depleted of ribosomal RNA and the RNA-seq libraries prepared with the Illumina TruSeq Stranded Total RNA kit following the manufacturer’s protocol. Amplified libraries were checked on a bioanalyzer 2100 and quantified with picogreen reagent. Libraries with distinct TruSeq adapter UDIndexes were multiplexed and, after cluster generation on FlowCell, were sequenced for 50 bases in paired-end mode with a Novaseq 6000 sequencer (40 × 10^6^ reads coverage).

### Protein extraction and western blot analysis

For Whole Cell Extracts -WCE- preparation, SUM159PT, BT549 and T47D cells were extracted with RIPA buffer [31]. Primary antibodies used were anti-NF-YA G-2 (sc-17753 Santa Cruz), anti-NF-YB (home-made), anti-NF-YC (home-made), anti-Vinculin (05-386 Sigma-Aldrich), anti-Myc (hybridomas 9E10). Secondary antibodies were peroxidase-conjugated anti-mouse (A4416 Sigma-Aldrich) and anti-rabbit (A6154 Sigma-Aldrich). Western blot experiments were performed on three independent biological replicates. Full and uncropped western blots are presented in Supplementary Fig. S8.

### Cellular assays

For size analysis, cells were fixed with 2% paraformaldehyde for 10’, washed with PBS, permeabilized by 0.1% TritonX-100 for 20’, blocked with 1% BSA for 20’, stained with Rhodamine Phalloidin 1:500 for 45’, washed three times and stained with Hoechst 33342 1:2000 for 5’. Pictures were taken at 40x magnification with Leica CTR6000 Fluorescent Microscope; areas of randomly chosen cells were collected and analysed with ImageJ 1.53.

Proliferation assays were performed by plating 2500 cells (SUM159PT) or 5000 (BT549) in 96-wells plates and counting every 24 h for 3 days, using the Hoechst 1:2000 with automated cell counting by High Content Screening. The number of cells were normalized on 4 h post-plating count.

For spheroids formation, 10^5^ SUM159PT and BT549 cells were plated in 10 cm non-coated plates; the medium was changed every 4 days and the data acquired after 14 days of growth. We collected images and analysed the shape and aggregation state.

For clonogenic assays in 2D Culture, 10^3^ SUM159PT and BT549 cells were plated in 10 cm dishes, medium changed after 7 days; after 14 days, plates were washed with PBS and fixed/stained with a Crystal Violet solution (Crystal violet 0.0005%, Formaldehyde 1%, Methanol 0.01%, in PBS) for 20’ at room temperature. We counted colonies with more than 50 cells.

For wound healing assay, cells were cultured in a 24-well culture plate for 24 h to 90%‒95% confluency. Wound line was created by scratching the plate with a 10 μl micropipette tip. Cells were washed with PBS and the average width of the gaps calculated from the image taken with a microscope. Invasion assay was performed Corning strain with 0.45 μm pore size membranes. Cells were resuspended in 100 μl of media without FBS and insulin and seeded at a density of 10^4^ cells per well. Membranes bottom sides were put in contact with 600 μl of complete media. After 24 h of culture, invasive cells were fixed and stained with crystal violet 0.2% solution, pictures taken at optical microscope (LEICA ICC50 W, 4×) and % of membrane area occupied by invasive cells was counted with the ImageJ software (LAS V4.9).

All biological data were obtained from at least three independent biological replicates, except for the BT549 transwell assay which were done in duplicate. Multiple comparisons were performed using the one-way ANOVA test.

### Zebrafish experiments

Zebrafish larvae of the AB strain were obtained through natural spawning of wild type adult fish. Our facility strictly complies with the relevant European (EU Directive, 2010/63/EU for animal experiments) and Italian (Legislative Decree No. 26/2014) laws, rules, and regulations, confirmed by the authorization issued by the municipality of Milan (PG 384983/2013). The procedures were carried out in accordance with the relevant guidelines and regulations.

48 h post-fertilization (hpf) anesthetized zebrafish larvae were microinjected in a fully randomized order with fluorescently labelled tumour cells as previously reported [59 and Reference therein]. Depending on the cells’ size, ~600 to ~1200 cells were injected directly into the perivitelline space. Briefly, SUM159PT and BT549, both controls and YAl-KO cells, were labelled with Hoechst 33342 solution (ThermoFisher) and resuspended in complete medium at a final concentration of 300 cells/nl, and 2 to 4 nl/larva were inoculated in a complete blind manner. Injected live larvae were immediately observed under a fluorescent microscope to ensure the presence of labelled tumour cells. At 24 h post-injection (hpi) the larvae were anesthetized, individually placed on a microscope slide, and the number of extravasated cells was counted using an inverted fluorescent microscope. At least two independent biological replicates were performed. The number (n) of larvae for each experiment is indicated in the Figure Legends.

### RNA-seq datasets and molecular classifications

We downloaded the RSEM scaled count data for TCGA BRCA and STAD cohorts from the http://firebrowse.org/ web page. As of June 2022, we found RNA-seq data on 1093 primary tumours, 7 metastatic and 35 non-tumour tissues for BRCA, 415 primary tumours and 35 normal adjacent tissue samples for STAD. We retrieved the FASTQ files associated to 51 BRCA cell lines from CCLE, and to six SUM159 cell line samples from Yang et al. [60]. For RBPs analyses, we acquired expression data from KD, OE and KO experiments [54, 61–64]. All FASTQ files were downloaded using the SRA Explorer website (https://sra-explorer.info/), and the accession codes are in Supplementary Table S4. From the FASTQ files, we calculated mRNA expression with RSEM 1.3.1.

For BRCA samples, the molecular classification was retrieved from Fougner et al. [28], and used as the training set for the DeepCC tool to classify samples previously unclassified or classified as Normal-like. As for STAD, we used the classification detailed in a previous work [25]. Finally, the molecular classification for all the analysed BRCA cell lines was derived from Prat et al. [65] and Dai et al. [66].

### Weighted gene co-expression network analysis

BRCA and STAD gene expression, as log_2_(TPM + 1), were used as input for weighted gene co-expression network analysis, employing the WGCNA R package (version 1.70-3) [67]. NF-YA gene-level expression was replaced with NF-YAl and NF-YAs isoforms expression, together with the NF-YAl/NF-YAs ratio (NF-YAr). The soft-thresholding powers for BRCA and STAD were set to 4 and 7, respectively, as suggested by the function *pickSoftThreshold*. The parameters for the *blockwiseModules* function responsible for building the networks and find gene modules were: *networkType* = “signed”, *maxBlockSize* = 30000, *minModuleSize* = 30, *reassignThreshold* = 0, and *mergeCutHeight* = 0.25.

### Gene expression analysis

Differential gene expression analysis of RNA-seq data was performed using the R package DESeq2 (version 1.30.1) [68]. We used expression fold change (FC) to denote upregulation or downregulation in BT549 and SUM159 YAl-KO clones versus parental cell lines. Log_2_FC, and the corresponding false discovery rate (FDR), were reported by the R package. FDR < 0.01 were set as inclusion criteria for DEG selection.

### Gene ontology and gene sets enrichment analyses

We used KOBAS 3.0 (http://kobas.cbi.pku.edu.cn/anno_iden.php) for Gene Ontology (GO) terms enrichment analysis using the ENTREZ gene IDs as input and filtering out terms with FDR ≥ 0.01. MSigDB Hallmark gene sets enrichment analyses were conducted with the Enrichr website (https://maayanlab.cloud/Enrichr). For heatmap of pathways, only gene sets with a Combined Score >1 in all four experiments were included.

### Z scores computation for signatures evaluation

We obtained Z scores from log_2_-transformed expression data (TPM) for each gene of the BRCA 7^th^ gene module and BRCA-STAD ratio signatures. Then, a median Z score computed across all genes of the signatures was associated to each sample.

### Analysis of clinical data

We retrieved TCGA BRCA and STAD patients Progression Free Interval -PFI- time records from the http://xena.ucsc.edu/ web page. We employed the Cutoff Finder tool [69] to find the optimal threshold for dichotomization of tumour samples based on the NF-YAr levels and PFI data. NF-YAr values >1 were set to 1, and survival analysis was performed according to the Kaplan–Meier analysis and a two-sided log-rank test.

### scRNA-seq cell type annotation and deconvolution

scRNA-seq experiments conducted on breast and gastric cancers [37, 38] were first annotated with the R package scTyper (version 0.1.0) [70] choosing the Nearest Template Prediction -NTP- as cell typing method. We used the same markers for the cell types included in the original papers, when present. For BRCA cancer fractions, Basal, Luminal (A and B), and HER2-enriched tumours markers were included, plus an EMT signature from Taube et al. [71]. Likewise, we added an EMT signature in STAD cell typing process. Supplementary Table S5 contains the complete list of markers selected for the scTyper analysis.

We used the package SCDC (version 0.0.0.9000) [72] to lead a bulk RNA-seq composition deconvolution and a predicted proportion for each cell type was associated to TCGA BRCA and STAD tumour samples. These samples were then divided according to NF-YAr deciles or molecular subtypes, and cell types average proportions were computed for these so-defined groups. Within each subtype, samples with high NF-YAr (fourth quartile) were considered separately from all others.

### Statistical analysis

All computational analyses were performed in the R programming environment (version 4.0.3), with the ggplot2, ggpubr, pheatmap, rstatix and tidyverse packages installed. Single comparisons between two groups were performed with the Wilcoxon rank-sum test (two-sided) or, in case of triplicates of two conditions, the Student *t*-test (two-sided).

## Supplementary information

Reproducibility checklist

Supplementary Figure S1

Supplementary Figure S2

Supplementary Figure S3

Supplementary Figure S4

Supplementary Figure S5

Supplementary Figure S6

Supplementary Figure S7

Supplementary Figure S8

Supplementary Table S1

Supplementary Table S2

Supplementary Table S3

Supplementary Table S4

Supplementary Table S5

## Data Availability

Rna-seq datasets generated and analysed during this study are included in this published article and its Supplementary Information files. The raw data are available at Gene Expression Omnibus (GEO) as GSE208088.
