# Supplementary figures and images for: NF-YAl drives EMT in Claudinlow tumours
Source: Cell Death Dis. 2023 Jan 28;14(1):65. doi: 10.1038/s41419-023-05591-9 (PMC9883497; doi:10.1038/s41419-023-05591-9)

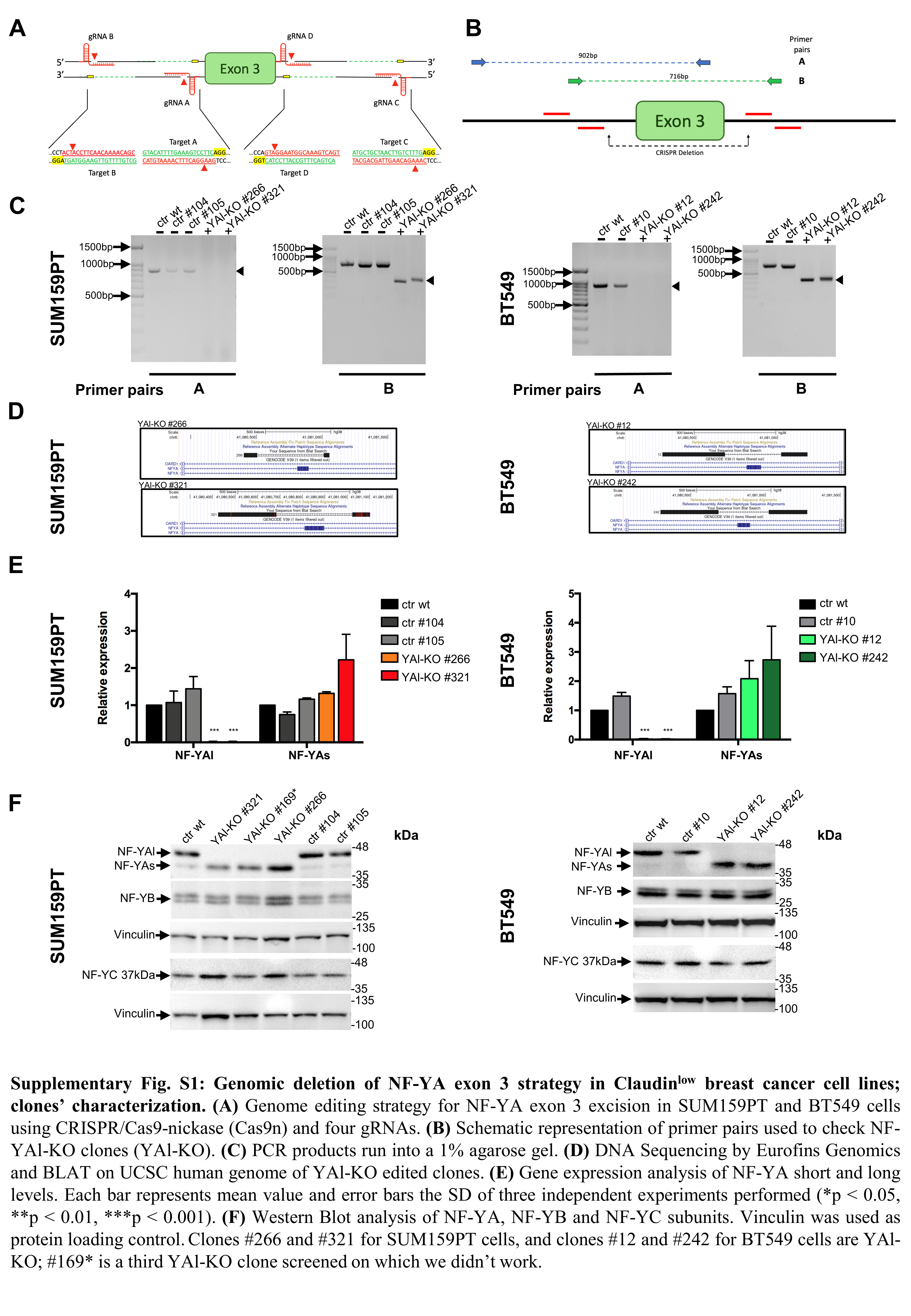

Supplement: Supplementary file 2 — Supplementary Figure S1 [file 41419_2023_5591_MOESM2_ESM.png]

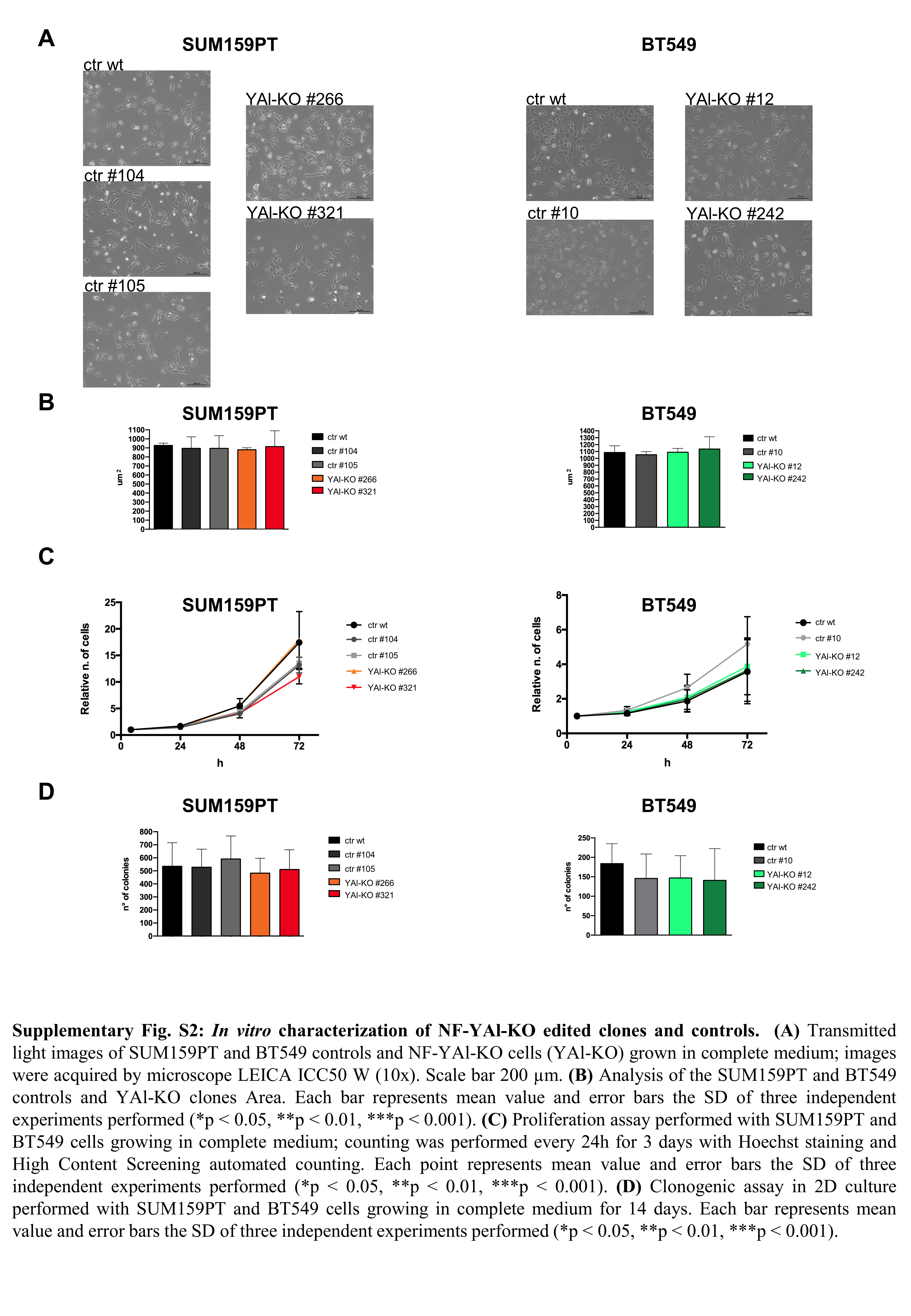

Supplement: Supplementary file 3 — Supplementary Figure S2 [file 41419_2023_5591_MOESM3_ESM.png]

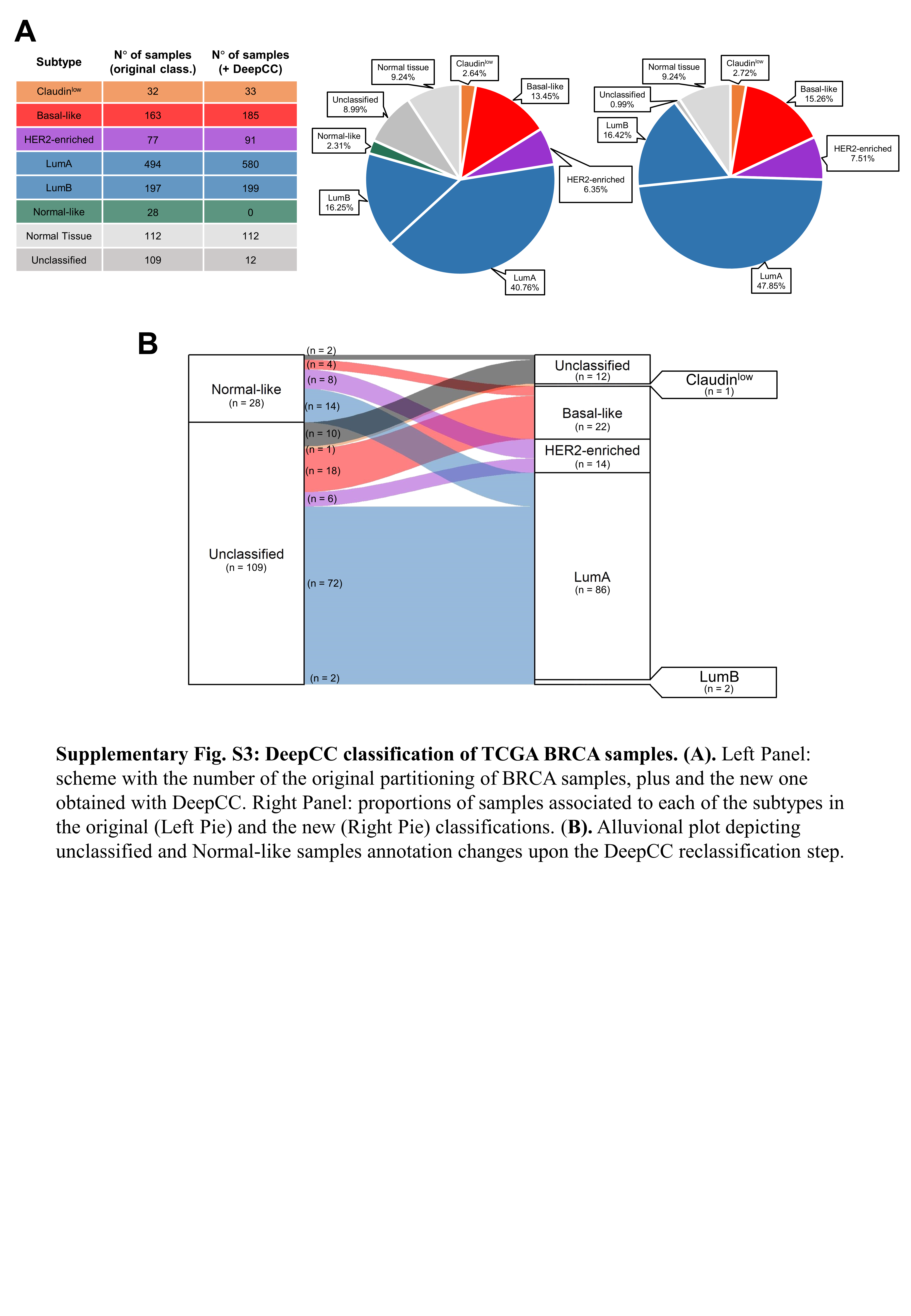

Supplement: Supplementary file 4 — Supplementary Figure S3 [file 41419_2023_5591_MOESM4_ESM.png]

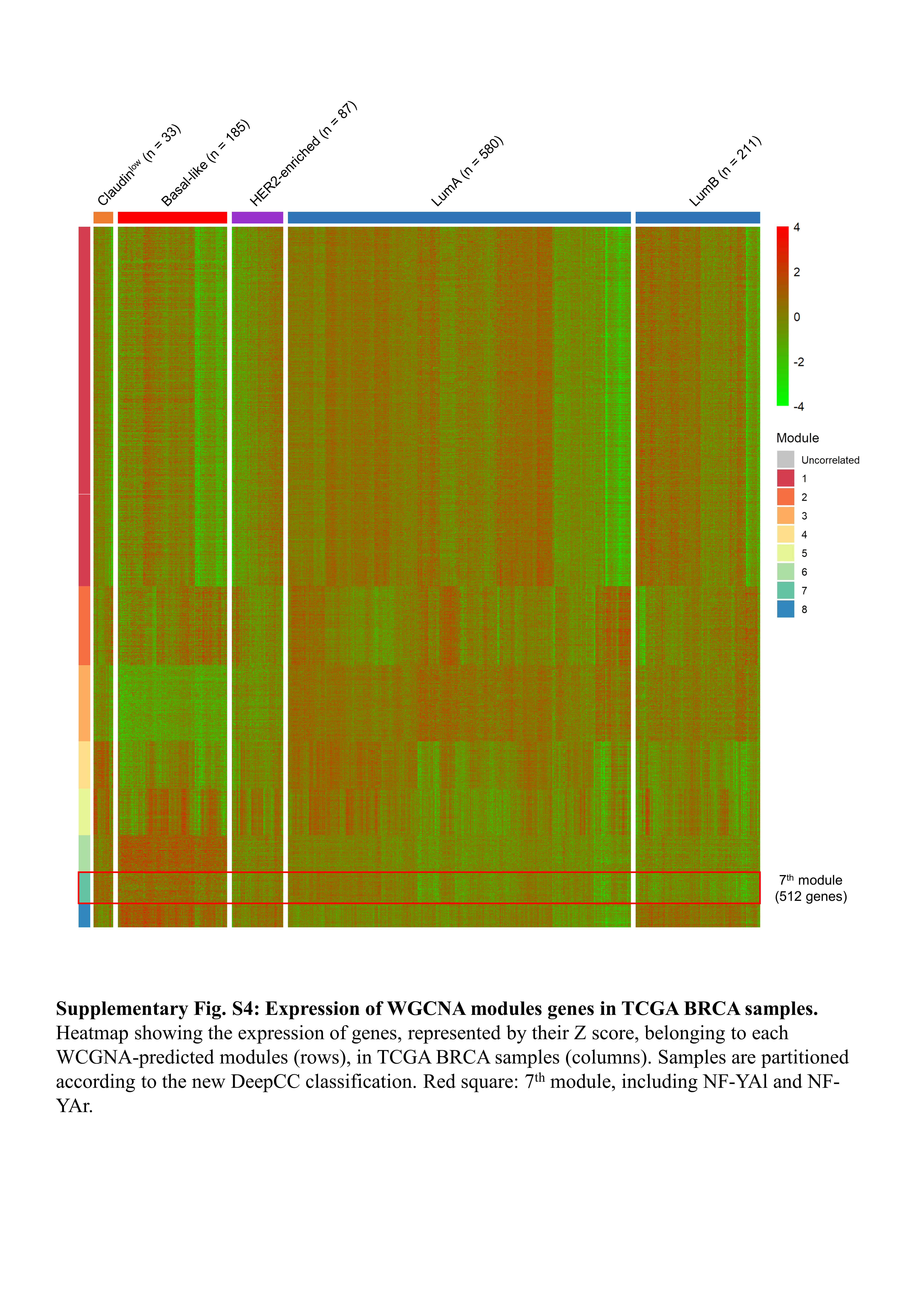

Supplement: Supplementary file 5 — Supplementary Figure S4 [file 41419_2023_5591_MOESM5_ESM.png]

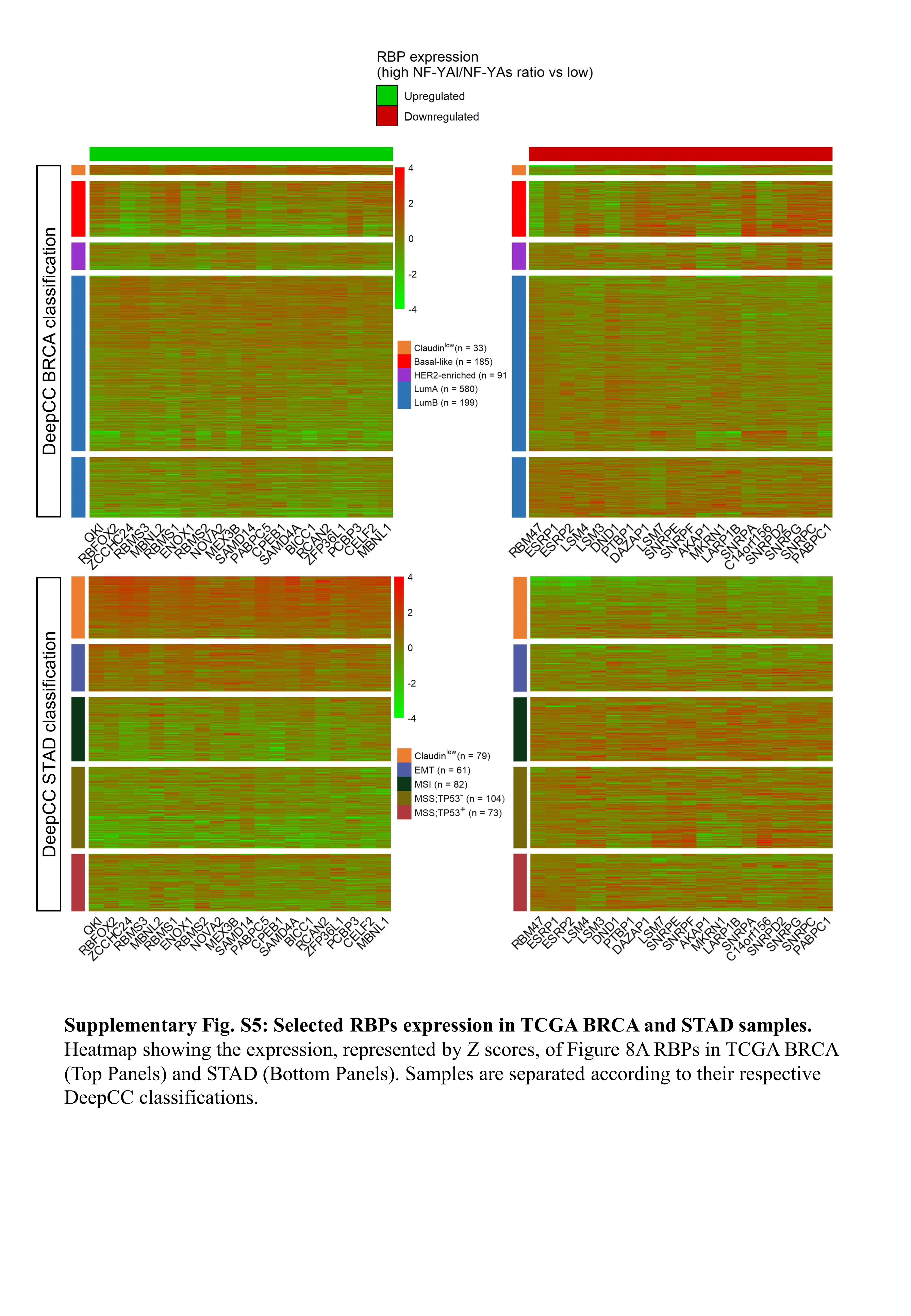

Supplement: Supplementary file 6 — Supplementary Figure S5 [file 41419_2023_5591_MOESM6_ESM.png]

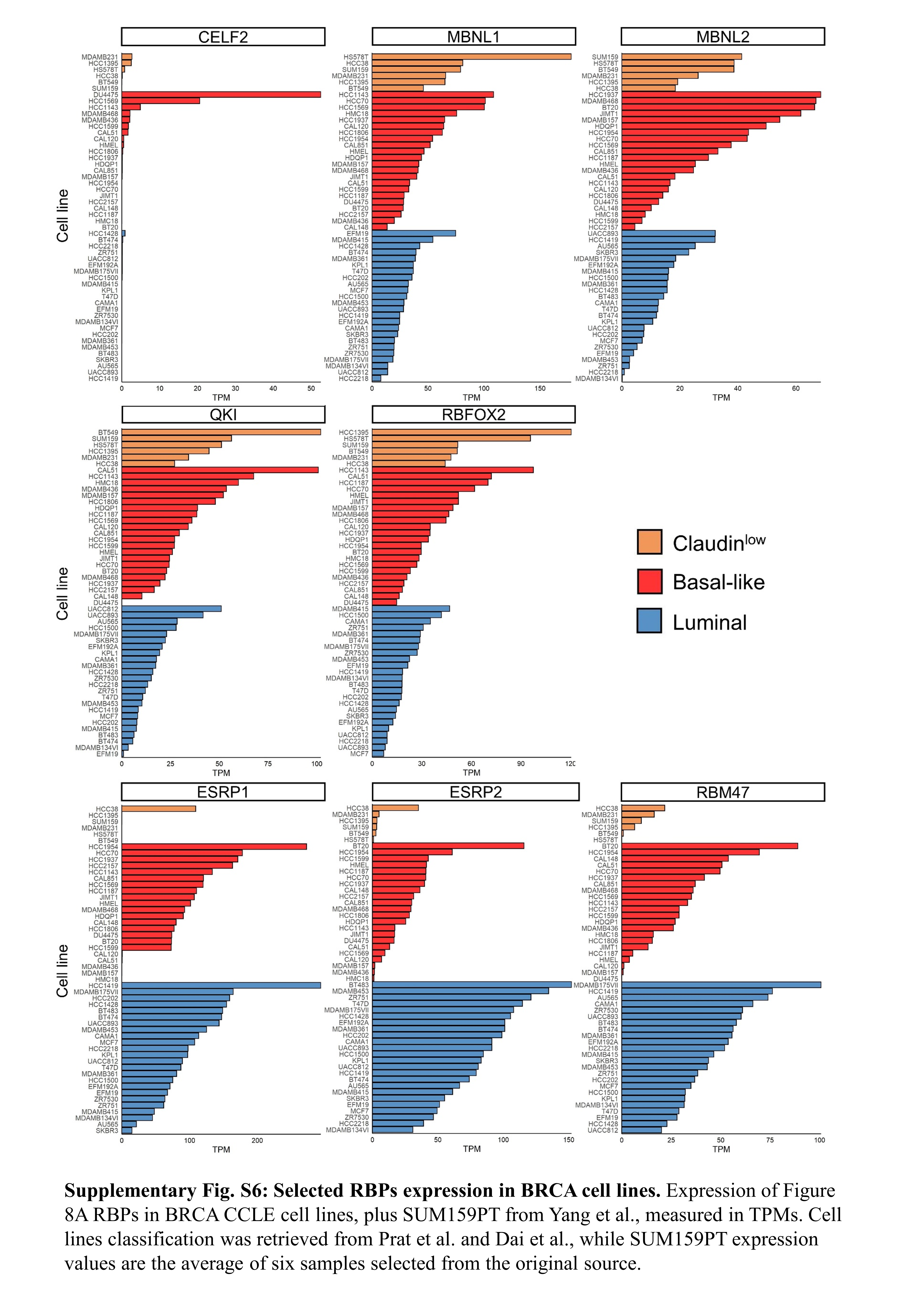

Supplement: Supplementary file 7 — Supplementary Figure S6 [file 41419_2023_5591_MOESM7_ESM.png]

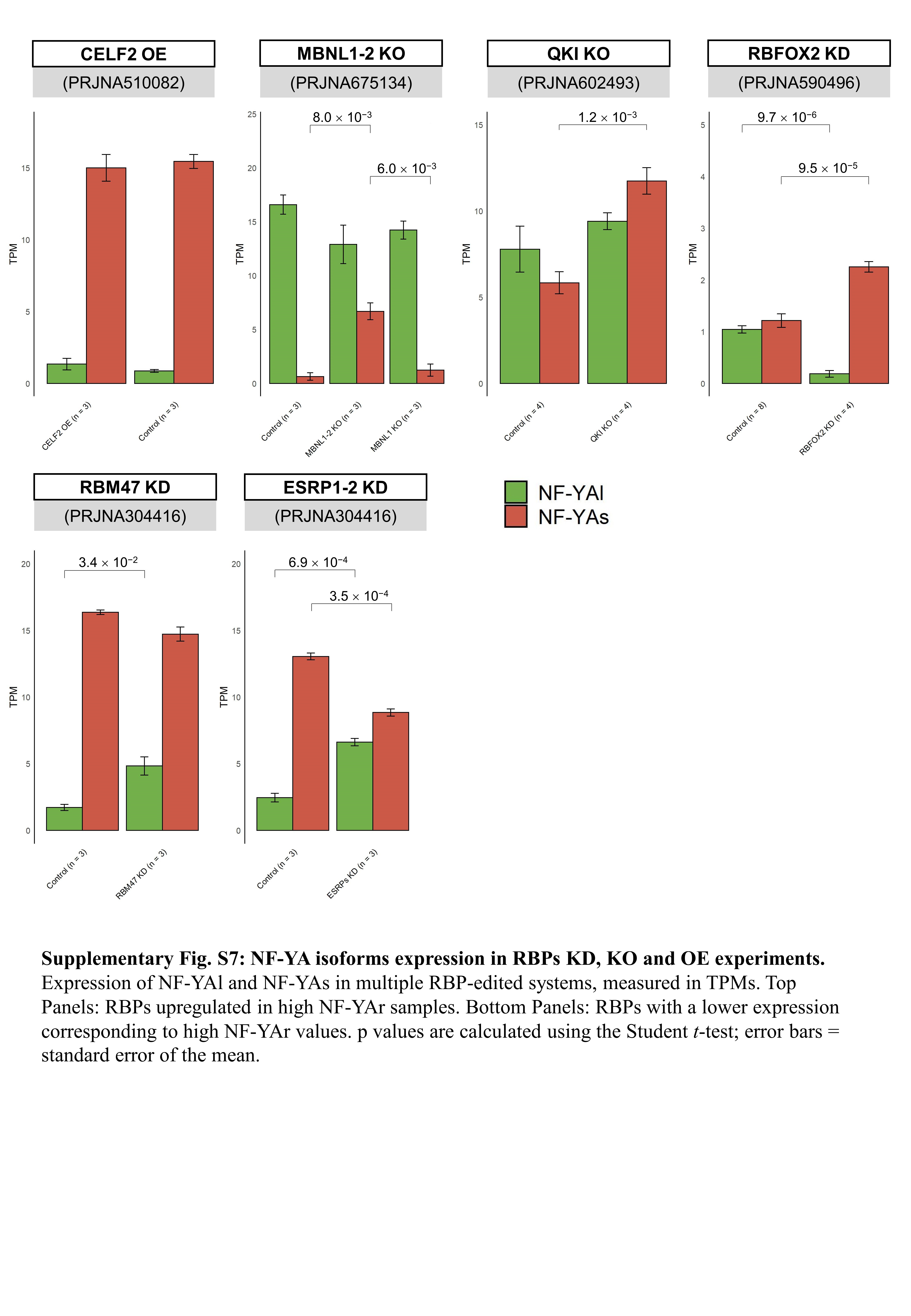

Supplement: Supplementary file 8 — Supplementary Figure S7 [file 41419_2023_5591_MOESM8_ESM.png]

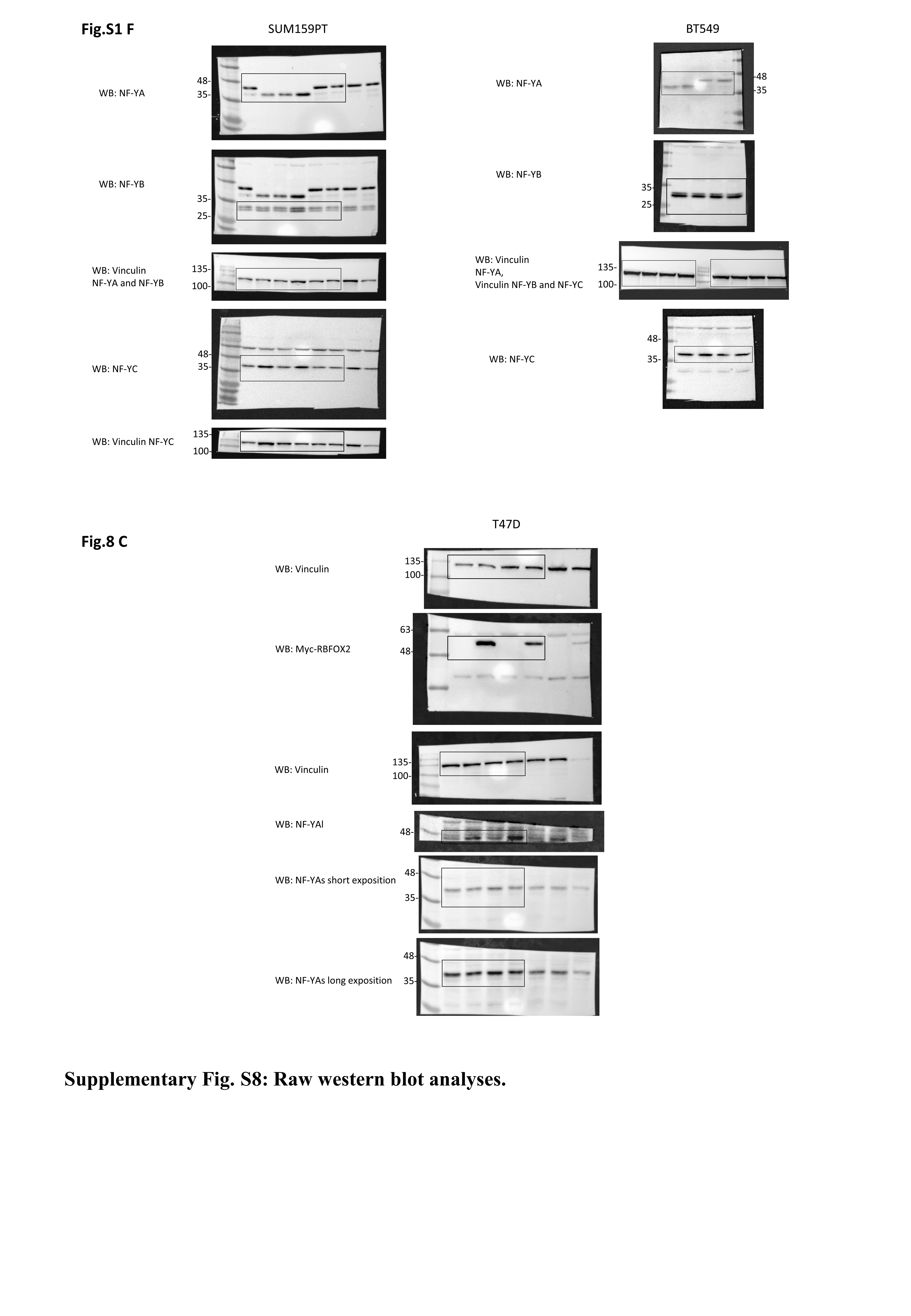

Supplement: Supplementary file 9 — Supplementary Figure S8 [file 41419_2023_5591_MOESM9_ESM.png]
